# Health behaviors in major chronic diseases patients: trends and regional variations analysis, 2008–2017, Korea

**DOI:** 10.1186/s12889-020-09940-7

**Published:** 2020-11-27

**Authors:** Young-Jee Jeon, Jeehee Pyo, Young-Kwon Park, Minsu Ock

**Affiliations:** 1 Department of Family Medicine, Ulsan University Hospital, University of Ulsan College of Medicine, Ulsan, Republic of Korea; 2 Department of Preventive Medicine, Ulsan University Hospital, University of Ulsan College of Medicine, 877 Bangeojinsunhwando-ro, Dong-gu, Ulsan, 44033 Republic of Korea; 3 Preventive Medicine Center, Ulsan University Hospital, Ulsan, Republic of Korea

**Keywords:** Chronic disease, Noncommunicable diseases, Health behavior, Smoking cessation, Alcohol drinking, Walking

## Abstract

**Background:**

Improving the health behaviors of those with chronic diseases such as hypertension and diabetes is important for disease management. Few in-depth studies have been conducted in Korea on the health behaviors of chronic disease patients. This study examined the health behaviors of chronic disease patients over time and compared them with those of the general population.

**Methods:**

Cross-sectional time-series data obtained from the Korea Community Health Survey from 2008 to 2017 were analyzed. Thirteen diseases were included in this analysis, namely, hypertension, diabetes, dyslipidemia, stroke, myocardial infarction, angina, osteoarthritis, osteoporosis, asthma, allergic rhinitis, atopic dermatitis, cataract, and depression. The current smoking rate, drinking rate, and the walking rate, which are leading health behaviors necessary for preventing chronic diseases, were analyzed by disease type. We compared patients’ health behaviors with those of the general population and identified regional variations.

**Results:**

Although the current overall smoking rate was seemingly declining, the overall monthly drinking and high-risk drinking rates were increasing. In 2017, patients experiencing depression symptoms had a higher smoking rate than did the general population; hypertension and diabetes patients had a higher risk-drinking rate than did the latter. The general population’s walking rate was highest. There were considerable variations by region among chronic disease patients.

**Conclusions:**

Chronic disease patients displayed worse health behaviors than those of the general population, in some instances. Rather than focusing only on chronic disease patients’ medication adherence, strategies must be devised to increase their smoking cessation rate, decrease their drinking rate, and increase their walking rate.

**Supplementary Information:**

The online version contains supplementary material available at 10.1186/s12889-020-09940-7.

## Background

Noncommunicable diseases (NCDs) are characterized as chronic, slow-progressing diseases, with a sharply increasing burden. NCDs contribute to 41 million deaths annually worldwide (71% of total deaths) [1]. Deaths from NCDs are expected to reach 52 million by 2030 [2]. Cardiovascular disease, cancer, chronic respiratory diseases, and diabetes are NCDs with sequentially the highest mortality rates [2]. Korea has a considerably high NCD burden; in 2015, the number of disability-adjusted life years (DALYs) due to NCDs was 25,683 for 100,000 people (87.1% of the total DALYs) [3].

To reduce the NCD burden, most public health policies aim to identify and manage modifiable health behaviors that can effectively reduce the number of deaths caused by NCDs [4]. The World Health Organization (WHO) compiled and introduced 16 cost-effective, easily implementable interventions as “best buys,” believing that these can reduce the 9.6 million global premature deaths caused by NCDs from 2018 to 2025. The following are modifiable behavioral risk factors targeted by interventions cited as “best buys”: reducing tobacco use, harmful alcohol use, an unhealthy diet, and physical inactivity [1].

While health behavior changes are known to control NCDs [5–11], poor health behaviors are not easy to improve [12]. However, since diagnosis with NCDs such as hypertension or diabetes is considered a health crisis, it may serve as a good opportunity (i.e., “wake-up call” or “teachable moment”) to correct poor health behaviors and prevent secondary diseases [13, 14]. While a previous study involving an educational intervention on managing health behaviors revealed improvement in chronic disease patients’ health behaviors [15], observational study findings on changes in health behaviors after NCD diagnoses are inconsistent [14, 16–26]. In many previous studies, NCD patients’ smoking rates decreased, among other health behaviors targeted by the WHO [14–17, 19, 20, 22, 23, 26]. While some previous studies reported decreased harmful alcohol drinking rate [14, 16, 26], the decrease in the drinking rate was lower than that of the smoking rate. Diagnoses did little to increase physical activity in most studies [14, 18, 19, 21, 23, 25]; one study reported decreased physical activity [16].

Most previous observational studies compared the health behaviors of newly diagnosed patients at 2 time points—before and after diagnosis—to examine the effect of a new NCD diagnosis on health behaviors [14, 16–20, 22–26]. Since most previous studies individually examined diseases such as hypertension [27, 28], diabetes [20, 23, 24], and cancer [22, 25], or only 4–5 NCDs [14, 16–19, 21, 26], they could not comprehensively clarify NCD patients’ health behaviors.

This study used large-scale, nationally representative data, to examine and compare the health behaviors of patients diagnosed with various chronic diseases with those of the general population. This study also analyzes annual trends to identify health behavior changes in the general population and chronic disease patients.

## Methods

### Source of data

Using cross-sectional time-series data of the Korea Community Health Survey from 2008 to 2017, this study compared the health behaviors of patients with major chronic diseases across 18 cities and provinces in Korea (for Sejong since 2012). The Korea Community Health Survey, conducted by the Korea Centers for Disease Control and Prevention since 2008, collects data from 253 community health centers in Korea on more than 220,000 adults aged ≥19 years (900 × 253 community health centers) to plan public health policies [29]. The survey population is all adults aged ≥19 years living in residential houses at each sample point and survey households were selected using systematic sampling by stratification into Tong/Ban/Lee (smallest administrative district units) and housing type. All adults ≥19 years living in the selected survey households were interviewed. The survey collects information about health behaviors such as smoking and drinking, medical history, healthcare use, health-related quality of life, and demographic characteristics.

### Definition of major chronic diseases

The Korea Community Health Survey checks for major chronic disease among participants. This analysis focused on hypertension, diabetes, dyslipidemia, stroke, myocardial infarction, angina, osteoarthritis, osteoporosis, asthma, allergic rhinitis, atopic dermatitis, cataract, and depression. A physician’s diagnosis confirmed a patient’s disease status. For instance, those answering “Yes” to the question, “Have you ever been diagnosed with diabetes by a physician?,” were defined as diabetes patients. Depression included not only whether participants had been physician-diagnosed, but also whether they had experienced depression symptoms. Those answering “Yes” to the question, “Have you ever felt so sad or devastated in the past 1 year that it affected your daily life for 2 weeks or longer consecutively?,” were defined as experiencing depression symptoms. The survey did not investigate annual occurrence of all the above conditions; Table 1 presents the survey’s annual tracking of diseases per year.

**Table 1:** Diseases included in this study by year

Diseases	2008	2009	2010	2011	2012	2013	2014	2015	2016	2017
Hypertension	O	O	O	O	O	O	O	O	O	O
Diabetes	O	O	O	O	O	O	O	O	O	O
Dyslipidemia (including hyperlipidemia)				O	O	O	O	O	O	O
Stroke	O	O	O	O	O	O	O		O	
Myocardial infarction	O	O	O	O	O	O				
Angina	O	O	O	O	O	O				
Myocardial infarction or angina							O		O	
Osteoarthritis	O	O	O	O	O	O	O	O	O	O
Osteoporosis	O	O	O	O	O	O			O	
Asthma	O	O	O	O	O	O		O		
Allergic rhinitis	O	O	O	O	O	O		O		
Atopic dermatitis	O	O	O	O	O	O		O		
Cataract	O	O	O	O	O	O				O
Depression (physician diagnosis)		O	O	O	O	O				
Depression (symptom experience)	O	O	O	O	O	O	O	O	O	O

### Health behaviors

This study analyzed the current smoking (total vs. male), drinking (monthly and high-risk drinking rates), and walking rates as leading health behaviors. The current smoking rate was defined by dividing the number of current smokers (every day or sometimes) among those who had smoked ≥5 packs (100 cigarettes) in their lifetime, by the total number of respondents. The monthly drinking rate was defined by dividing the number of those who had drunk once per month in the past year by the total number of respondents. The high-risk drinking rate was defined by dividing the number of those who drank 7 (men) or 5 glasses (women) in one occasion ≥2 times a week in the past year, by the number of those who drank in the past year. The walking rate was defined by dividing the number of those who walked 30 min daily for ≥5 days per week in the past week, by the total number of respondents.

### Analysis

With descriptive analysis, the health behaviors of patients per disease were organized by year and region and compared with those of the general population. The general population was operationalized as all the participants in the Korea Community Health Survey. In addition, we obtained each standard deviation (SD) for health behaviors in 18 cities and provinces, to identify regional variations.

To examine any significant changes in health behaviors, joinpoint regression analysis was performed [30]. We permitted no more than 1 joinpoint, calculated the annual percentage change (APC) and average APC (AAPC) (when the number of joinpoints was 1), and presented the *p*-value for each. Stata/SE13.1 (StataCorp, Texas, TX) was used for descriptive analysis. For joinpoint regression, we used the Joinpoint Regression Program version 4.6.0 (US National Cancer Institute, Bethesda, MD, USA).

## Results

**Fig. 1:**
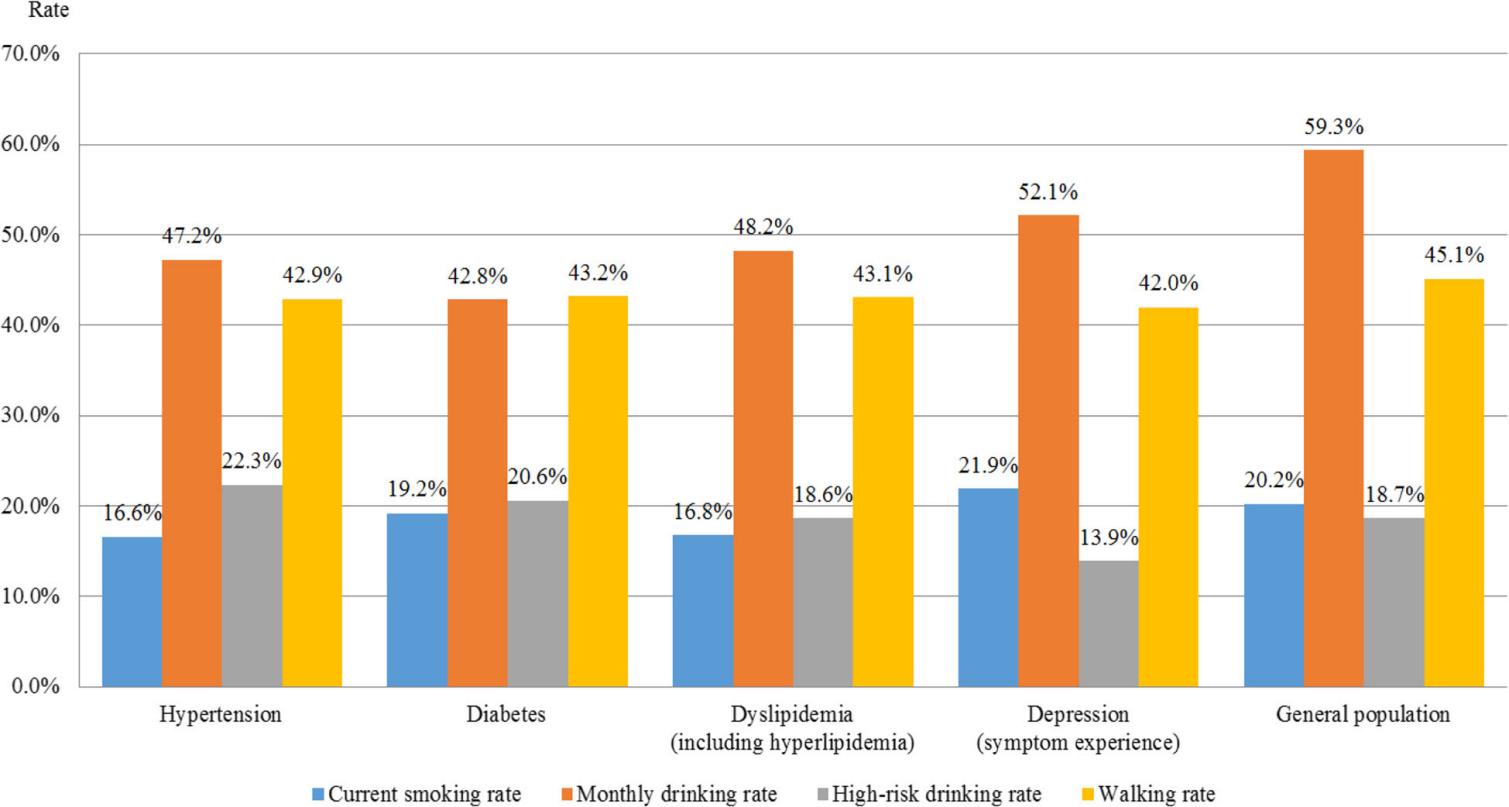
Comparison of the health behaviors of patients with major chronic diseases (as of 2017)

Overall, chronic disease patients and the general population did not show major health behavior differences. In 2017, while the general population’s current smoking rate was 20.2%, those of hypertension and diabetes patients were 16.6 and 19.2%, respectively (Fig. 1). The current smoking rate of patients experiencing depression symptoms was 21.9%—higher than that of the general population. The general population’s monthly drinking rate was highest (59.3%), with that of patients experiencing depression symptoms at > 50%. Overall, high-risk drinking rates have been on the rise since 2010, in particular, the high-risk drinking rates of hypertension and diabetes patients were 22.3 and 20.6%, respectively—higher than that of the general population (18.7%). The walking rate was highest in the general population (45.1%) and lowest (42.0%) in patients experiencing depression symptoms.

### Current smoking rate trends

**Fig. 2:**
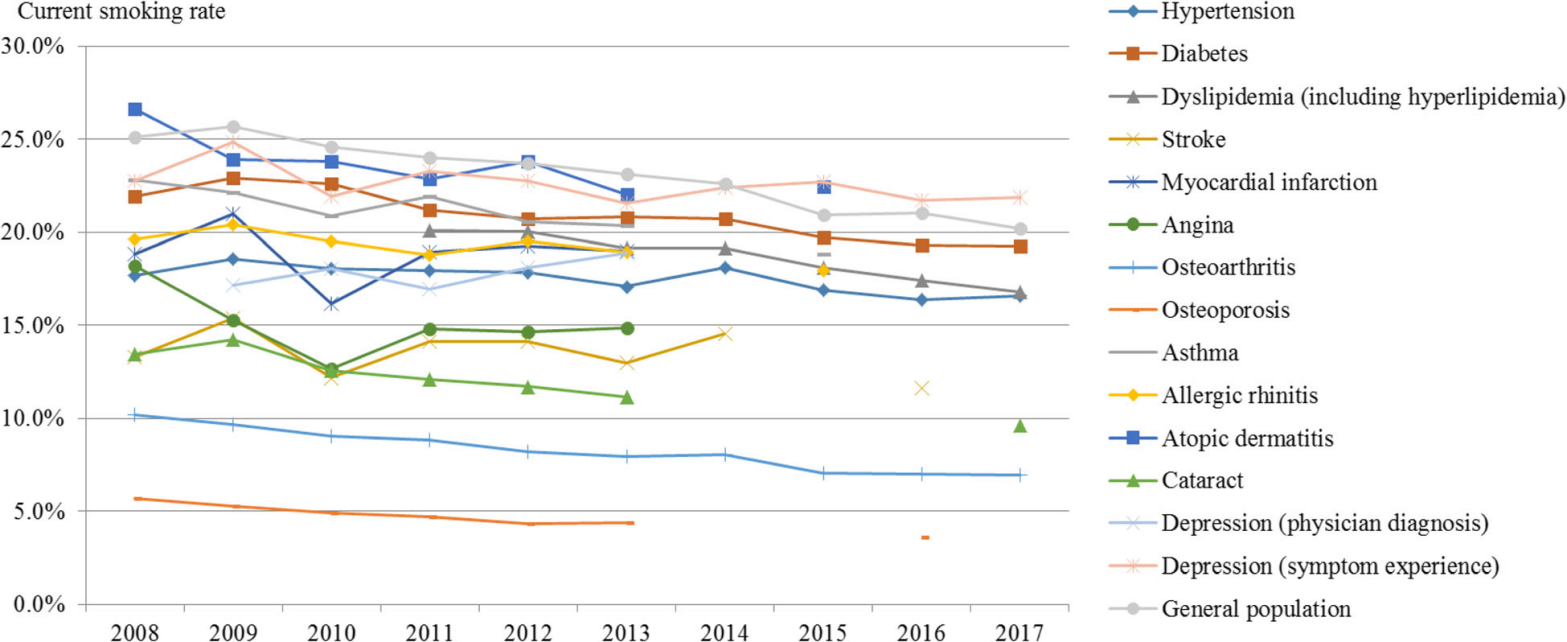
Trends in the current smoking rate (total) by disease

The current overall smoking rate showed a declining trend (Fig. 2, Supplement 1). The general population’s current smoking rate was 25.1% in 2008, decreasing to 20.2% in 2017 (APC: − 2.63, *p*-value: < 0.001). That among hypertension patients decreased from 17.7% in 2008 to 16.6% in 2017, but the decrease was smaller than that in the general population (APC: − 1.07, *p*-value: 0.006). By contrast, the current smoking rate among depression patients increased from 17.2% in 2009 to 18.9% in 2013 (APC: 1.90, *p*-value: 0.195).

**Fig. 3:**
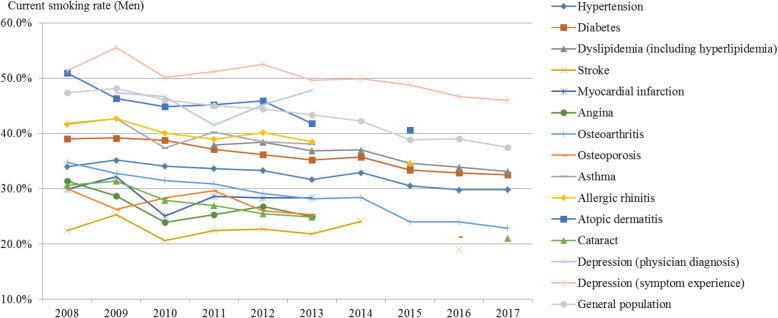
Trends in the current smoking rate (male) by disease

The current male smoking trend was similar to the overall trend (Fig. 3, Supplement 1). In the general population, the current male smoking rate declined from 47.3% in 2008 to 37.4% in 2017 (APC: − 2.79, *p*-value: < 0.001). In diabetes patients, this rate decreased from 39.0% in 2008 to 32.5% in 2017, but the decrease was smaller than that in the general population (APC: − 2.25, *p*-value: < 0.001).

### Drinking rate trends

**Fig. 4:**
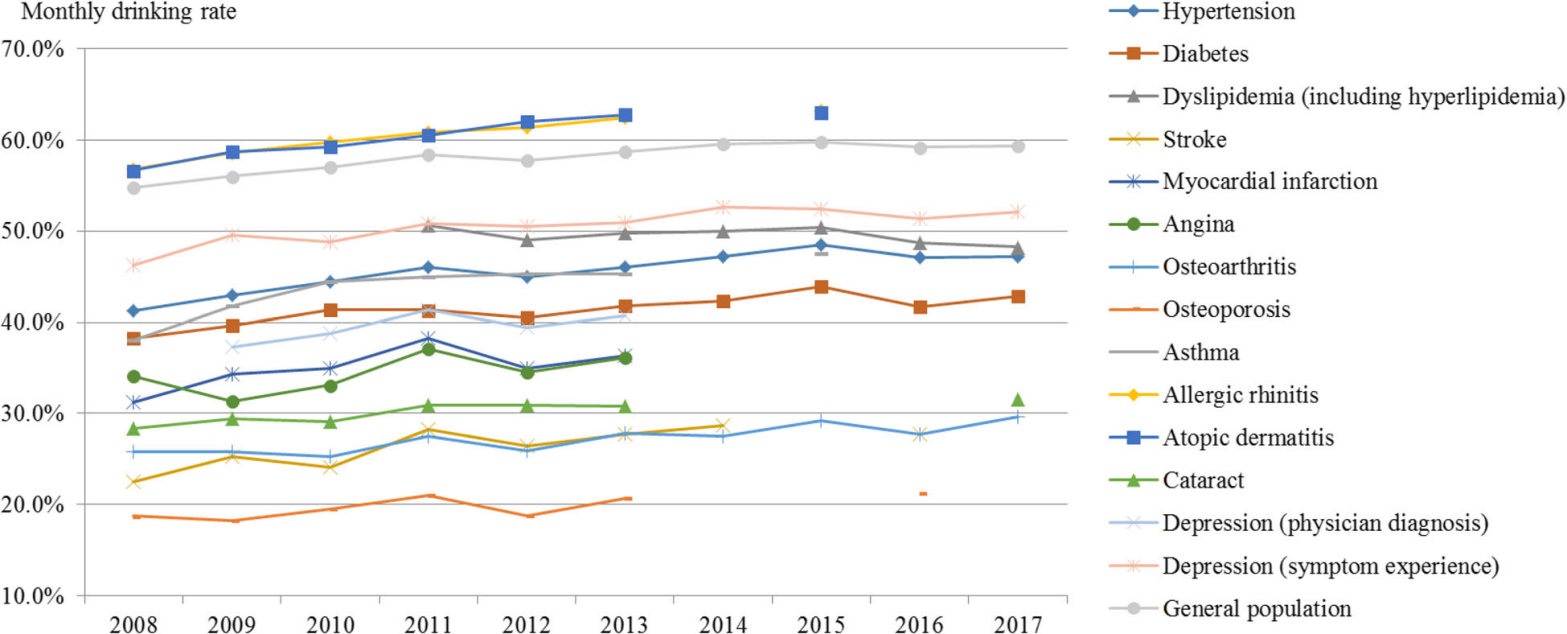
Trends in the monthly drinking rate by disease

The overall monthly drinking rate showed an increasing trend (Fig. 4, Supplement 1). The monthly drinking rate of the general population was 54.8% in 2008, increasing to 59.3% in 2017 (AAPC: 0.95, *p*-value: < 0.001). That of hypertension patients increased from 41.3% in 2008 to 47.2% in 2017; the increase was larger than that in the general population (AAPC: 1.22, *p*-value: 0.056). The monthly drinking rate of asthma patients showed the largest increase (AAPC: 3.01, *p*-value: < 0.001) from 38.9% in 2008 to 47.6% in 2015. It also increased for patients with cerebrovascular and cardiovascular diseases such as stroke, myocardial infarction, and angina.

**Fig. 5:**
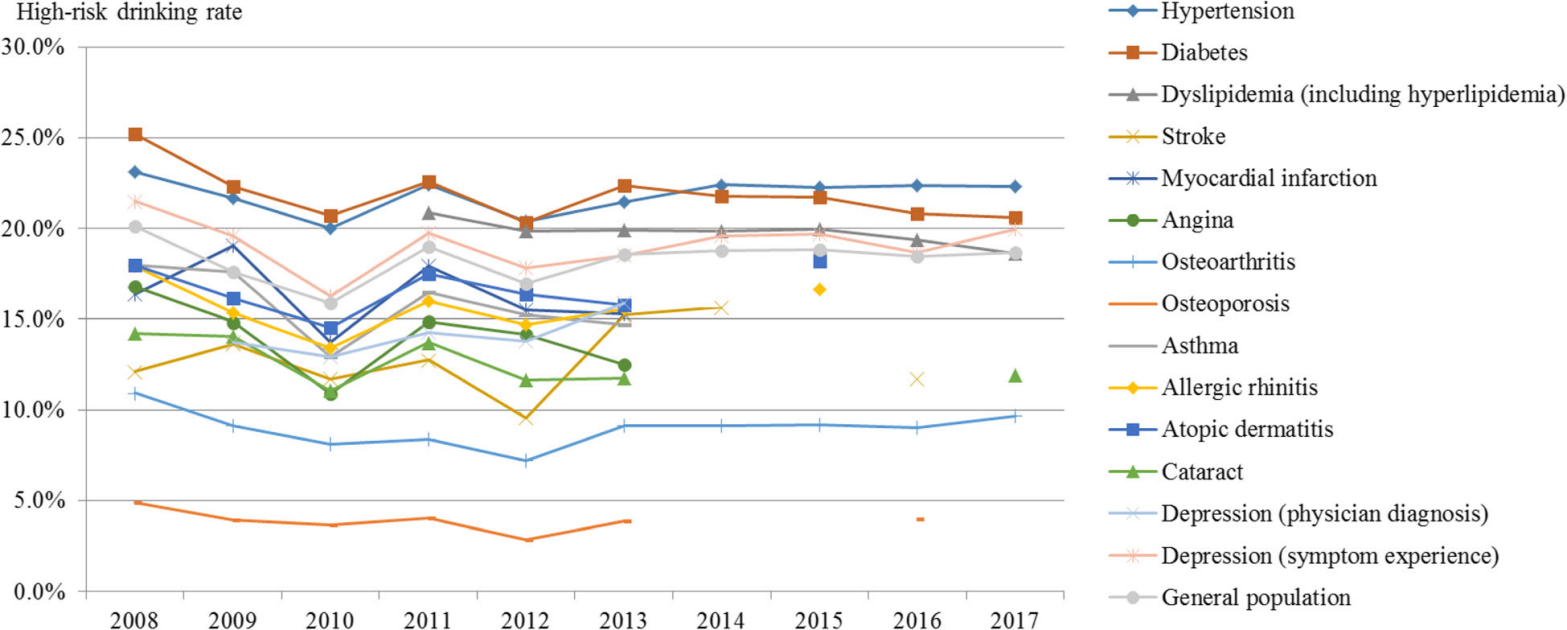
Trends in the high-risk drinking rate by disease

The overall high-risk drinking rate showed an increasing trend, except from 2008 (Fig. 5, Supplement 1). The general population’s high-risk drinking rate was 17.6% in 2009, increasing to 18.7% in 2017 (APC: 0.38, *p*-value: 0.634). That among hypertension patients increased from 21.6% in 2009 to 22.3% in 2017; the increase was as large as that in the general population (APC: 0.31, p-value: 0.566). While the high-risk drinking rate among diabetes patients decreased from 22.3% in 2009 to 20.6% in 2017 (APC: − 1.25, *p*-value: 0.069), it remained higher than that of the general population. That among patients experiencing depression symptoms showed little change and was higher than that of the general population (APC: − 0.01, *p*-value: 0.995).

### Trends in the walking rate

**Fig. 6:**
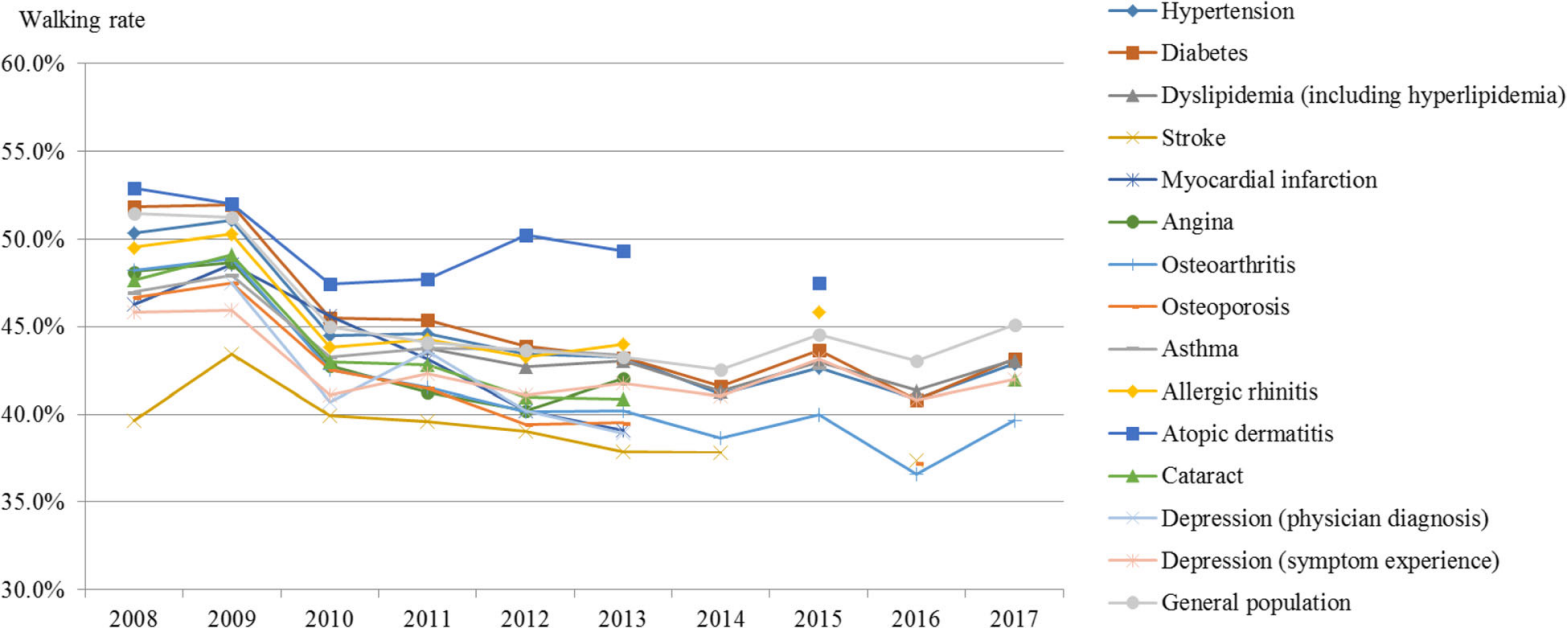
Trends in the walking rate by disease

The overall walking rate showed a declining trend (Fig. 6, Supplement 1). The general population’s walking rate was 51.4% in 2008, decreasing to 45.1% in 2017 (AAPC: − 1.90, p-value: 0.026). Diabetes patients’ walking rate decreased from 51.8% in 2008 to 43.2% in 2017; the decrease was larger than that in the general population (AAPC: − 2.36, p-value: 0.015). The walking rate of patients with depression was 47.5% in 2009, decreasing to 38.9% in 2013 (APC: − 4.04, p-value: 0.090).

### Variation by region

Regional variations were larger in the health behaviors of chronic disease patients than those of the general population (Supplement 1). As of 2017, Incheon (22.2%) had the highest current smoking rate for the general population, and Sejong, the lowest rate (18.3%) (SD: 1.1%). Jeju (27.9%) had the highest current smoking rate for patients experiencing depression symptoms, and Sejong (16.0%), the lowest rate (SD: 2.9%). As of 2017, Jeju (40.6%) had the highest current male smoking rate for the general population, and Sejong (34.8%), the lowest rate (SD: 1.6%). In the same year, Busan (36.0%) had the highest current smoking rate for diabetes patients, and Gwangju (25.5%), the lowest rate (SD: 2.8%).

As of 2017, Sejong (62.2%) had the highest monthly drinking rate for the general population, and Jeonbuk (50.7%), the lowest rate (SD: 3.2%). In the same year, Sejong (54.1%) had the highest monthly drinking rate for hypertension patients, and Jeonnam (35.9%), the lowest rate (SD: 4.5%). As of 2017, Gangwon (22.5%) had the highest high-risk drinking rate for the general population, and Gwangju (15.7%), the lowest rate (SD: 2.1%). In the same year, Jeju (25.9%) had the highest high-risk drinking rate for diabetes patients, and Jeonnam (15.1%), the lowest rate (SD: 3.2%).

Unlike other health behaviors, the walking rate showed larger regional variations in the general population. As of 2017, Seoul (61.1%) had the highest walking rate for the general population, and Gangwon (32.4%), the lowest rate (SD: 7.5%). In the same year, Seoul (57.6%) had the highest walking rate in hypertension patients, and Jeju (29.5%), the lowest rate (SD: 7.3%).

## Discussion

This study used the Korea Community Health Survey to examine the health behaviors of patients with major chronic diseases with regard to smoking, drinking, and physical activity, and compared these with those of the general population. We analyzed these trends from 2008 to 2017. Patients with major chronic diseases did not show notably better health behaviors; some showed worse health behaviors than did the general population. Although the smoking rate decreased, the decrease in patients with major chronic diseases was smaller than that in the general population. Regional variations were larger in the health behaviors of patients with major chronic diseases, compared to those of the general population.

Improved health behaviors can prevent NCDs and reduce their recurrence risk and severity, improve health-related quality of life, and extend life expectancy [31, 32]. Hence, the importance of health behaviors should be conveyed to the general population, to improve NCD patients’ health behaviors. Given the general difficulty of changing health behaviors [12], effective strategies should be developed, and resources assigned accordingly. Rather than focusing only on chronic disease patients’ medication adherence, strategies must be devised to encourage smoking cessation, reduce alcohol consumption, and increase patients’ walking rate. This study holds significance, as it presented empirical evidence to enable goal setting, so as to improve chronic disease patients’ health behaviors, and provided reasons for such improvement.

Smoking cessation has been highlighted for its potential to prevent or stabilize NCDs [33]. In particular, an NCD diagnosis is expected to serve as an important opportunity for smokers to change their smoking behaviors; in fact, the smoking rate has shown decline after a stroke [34], cancer [22], and diabetes [23] diagnosis. However, findings that > 3 out of 4 patients still smoked 2 years after a stroke diagnosis, and that only 20% of patients with lung diseases quit smoking within 2 years [14], suggest that a diagnosis does not necessarily translate into smoking cessation. In this study, the current smoking rate of hypertension (16.6% as of 2017) and diabetes patients (19.2% as of 2017) did not differ substantially from that of the general population (20.2% as of 2017), suggesting a need for more active treatment for smoking cessation for chronic disease patients.

We assumed that smoking was particularly concerning in patients with mental disorders. Smoking increased only among depression patients (17.2% in 2009 to 18.9% in 2013) in this study, and the current smoking rate of patients experiencing depression symptoms (21.9% as of 2017) was higher than that of the general population. These results were consistent with those of previous studies, showing a higher smoking rate among patients with mental disorders, that these patients accounted for a higher proportion of smokers, and that they were less likely to quit smoking successfully [35, 36]. A study reported that smoking cessation led to depression [37], suggesting that it might be better to let depressed patients smoke, thereby discouraging the smoking cessation recommendation to these patients. However, given mounting evidence on how to treat smoking patients with mental disorders [38], and the latest empirical evidence suggesting that smoking cessation actually decreases depression [39, 40], it is worth focusing more on smoking cessation among patients with conditions such as depression.

This study also found a higher drinking rate in chronic disease patients. While chronic disease patients’ monthly drinking rate was lower than that of the general population, those with cerebrovascular and cardiovascular diseases displayed a higher rate. Of more concern was that the high-risk drinking rate of patients with depression symptoms, hypertension, and diabetes was higher than that of the general population. Drinking is generally associated various NCDs including cancer, hypertension, hemorrhagic stroke, and liver diseases [41]. While small amounts of alcohol reportedly show preventative effects on diabetes [42], they also increase the risk of chronic diseases such as atrial fibrillation [43] and cataracts [44]. We cannot recommend small alcohol amounts to prevent some diseases, at the risk of others developing, nor can we recommend such to current chronic disease patients. Moreover, given drinking’s high correlation with smoking [45], we cannot rule out the possible effect of drinking on smoking behaviors.

More focus should be directed towards drinking in Korea, considering increases in the monthly and high-risk drinking rates in both the general population and patients with major chronic diseases. A permissive drinking culture has developed in Korea, with drinking considered to represent non-verbal communication, demonstrate a sense of community, and serve as a bridge between work and leisure. This permissive culture may have contributed to 6 out of 10 Korean adults drinking once or more per month. The higher the average drinking rate, the likelier problematic drinking is. Thus, drinking reduction strategies for the general population and chronic disease patients are necessary. To reduce drinking among the latter, physicians should assess their drinking patterns at each consultation, inform them of the harm associated with drinking, and actively recommend drinking cessation.

Physical activity is important for chronic disease management. The benefits of physical activity for hypertension and diabetes patients are well known; regular physical activity is recommended in the treatment guidelines for these diseases [46, 47]. Furthermore, exercise’s confirmed depression alleviation effect [48] has prompted its recommendation to depression patients. Moreover, despite exercise seeming somewhat risky for diseases like myocardial infarction, exercise-centered cardiac rehabilitation improves quality of life and reduces the mortality rate for myocardial infarction [49]. Therefore, exercise must be recommended to chronic disease patients, and the level of physical activity (e.g., the walking rate) must be evaluated as an indicator. The current study showed that the walking rate of patients with major chronic diseases was lower than that of the general population; there were annual declining trends in the walking rates of the general population and patients with major chronic diseases. Although the obesity rate in Korea is lower than that in other countries, a declining walking rate may be related to a consistently increasing obesity rate. Programs for increasing the walking rate in chronic disease patients and the general population are necessary.

Notably, although variations are affected by the sample sizes, regional variations in health behaviors were larger in chronic disease patients than in the general population. For instance, the standard deviation for the general population’s current smoking rate was 1.1% as of 2017, and 2.9% for patients experiencing depression symptoms. While for the former, the standard deviation for the monthly drinking rate was 3.2%, it was 4.5% for hypertension patients. Although the life expectancy gap between different regions in Korea has narrowed, a quality-adjusted life expectancy gap of 4.6 years has been shown between regions [50]. While many factors such as unequal health resources and income gaps ostensibly contribute towards this gap, a regional gap in health behaviors may also explain this phenomenon. More studies are needed to explain the regional health behavior gap.

This study had limitations. First, changes in health behaviors, based on a chronic disease diagnosis, were not examined due to the nature of this study’s data source. Future studies could use panel or patient cohort data to examine changes in chronic disease patients’ health behaviors over time before and after diagnosis. Second, chronic diseases were self-reported. The accuracy of these self-reports of physician diagnosis and health behaviors used in the Korea Community Health Survey, cannot be determined. A follow-up study would need to confirm the diagnoses and the health behaviors in relation to physical assessments or healthcare use data. Third, although this study focuses on patients with 13 chronic diseases, cancer—one of the leading NCDs—was excluded. Studies could analyze annual trends and regional variations in cancer patients’ health behaviors, using this study’s methodology. In addition, the gender differences in the smoking rate or health behavior of chronic disease patients could be a good research topic which we will consider in the future.

## Conclusions

This study using large-scale, nationally representative data confirmed that patients with major chronic diseases did not show particularly better health behaviors than did the general population, with some even displaying worse health behaviors. This study will enable goal setting aimed at improving chronic disease patients’ health behaviors, in particular in regional level. Goal setting and management, and strategizing in this regard, are important not only regarding chronic disease patients’ medication adherence, but also health behaviors such as smoking cessation, alcohol abstinence, and physical activity. Physicians who diagnose chronic disease patients could also implement strategies that identify and improve patients’ health behaviors. Furthermore, considering the difficulty of improving the general population’s health behaviors, an effective public health approach may be necessary in this regard, instead of reliance on individuals to improve their health behaviors.

## Supplementary Information

**Additional file 1.**

## Data Availability

All data generated or analysed during this study are included in this published article and its supplementary information files. The raw data used for this study analysis (Korea Community Health Survey) can be obtained from the following URL (Korean version): https://chs.cdc.go.kr/chs/main.do.

## Abbreviations

NCDs: Noncommunicable diseases
DALYs: Disability-adjusted life years
WHO: World Health Organization
APC: Annual percentage change
AAPC: Average annual percentage change
SD: Standard deviation

## References

[1] Noncommunicable disease country profiles 2018 [Internet]. Geneva: World Health Organization; 2018. Available from: https://www.who.int/publications/i/item/9789241514620 Accessed 1 Jan 2020.

[2] Global Health Estimates Summary Tables (2018). Projections of Deaths by Cause, Age and Sex by World Bank Income Category and WHO Region.

[3] Kim YE, Park H, Jo MW, Oh IH, Go DS, Jung J (2019). Trends and patterns of burden of disease and injuries in Korea using disability-adjusted life years. J Korean Med Sci.

[4] Kim HC, Oh SM (2013). Noncommunicable diseases: current status of major modifiable risk factors in Korea. J Prev Med Public Health.

[5] Grundy SM, Stone NJ, Bailey AL, Beam C, Birtcher KK, Blumenthal RS (2019). 2018 AHA/ACC/AACVPR/AAPA/ABC/ACPM/ADA/AGS/APhA/ASPC/NLA/PCNA guideline on the Management of Blood Cholesterol: a report of the American College of Cardiology/American Heart Association task force on clinical practice guidelines. Circulation..

[6] Coronado-Zarco R (2019). Olascoaga-Gomez de Leon a, Garcia-Lara a, Quinzanos-Fresnedo J, Nava-Bringas TI, Macias-Hernandez SI. Nonpharmacological interventions for osteoporosis treatment: systematic review of clinical practice guidelines. Osteoporos Sarcopenia.

[7] American Diabetes Association (2019). 5. Lifestyle Management: Standards of Medical Care in Diabetes-2019. Diabetes Care.

[8] Yeh GY, Horwitz R (2017). Integrative medicine for respiratory conditions: asthma and chronic obstructive pulmonary disease. Med Clin North Am.

[9] Riegel B, Moser DK, Buck HG, Dickson VV, Dunbar SB, Lee CS (2017). Self-Care for the Prevention and Management of Cardiovascular Disease and Stroke: A Scientific Statement for Healthcare Professionals From the American Heart Association. J Am Heart Assoc.

[10] Barros MBA, Lima MG, Azevedo RCS, Medina LBP, Lopes CS, Menezes PR (2017). Depression and health behaviors in Brazilian adults - PNS 2013. Rev Saude Publica.

[11] Chobanian AV, Bakris GL, Black HR, Cushman WC, Green LA, Izzo JL (2003). The seventh report of the joint National Committee on prevention, detection, evaluation, and treatment of high blood pressure: the JNC 7 report. Jama..

[12] Kelly MP, Barker M (2016). Why is changing health-related behaviour so difficult?. Public Health.

[13] McBride CM, Puleo E, Pollak KI, Clipp EC, Woolford S, Emmons KM (2008). Understanding the role of cancer worry in creating a "teachable moment" for multiple risk factor reduction. Soc Sci Med.

[14] Newsom JT, Huguet N, McCarthy MJ, Ramage-Morin P, Kaplan MS, Bernier J (2012). Health behavior change following chronic illness in middle and later life. J Gerontol B Psychol Sci Soc Sci.

[15] de Vries TI, Dorresteijn JAN, van der Graaf Y, Visseren FLJ, Westerink J (2019). Heterogeneity of treatment effects from an intensive lifestyle weight loss intervention on cardiovascular events in patients with type 2 diabetes: data from the look AHEAD trial. Diabetes Care.

[16] van Gool CH, Kempen GI, Penninx BW, Deeg DJ, van Eijk JT (2007). Chronic disease and lifestyle transitions: results from the longitudinal aging study Amsterdam. J Aging Health.

[17] Keenan PS (2009). Smoking and weight change after new health diagnoses in older adults. Arch Intern Med.

[18] King DE, Mainous AG (2009). 3rd, Carnemolla M, Everett CJ. Adherence to healthy lifestyle habits in US adults, 1988-2006. Am J Med.

[19] Newson JT, Huguet N, Ramage-Morin PL, McCarthy MJ, Bernier J, Kaplan MS (2012). Health behaviour changes after diagnosis of chronic illness among Canadians aged 50 or older. Health Rep.

[20] Chong S, Ding D, Byun R, Comino E, Bauman A, Jalaludin B (2017). Lifestyle changes after a diagnosis of type 2 diabetes. Diabetes Spectr.

[21] Dontje ML, Krijnen WP, de Greef MH, Peeters GG, Stolk RP, van der Schans CP (2016). Effect of diagnosis with a chronic disease on physical activity behavior in middle-aged women. Prev Med.

[22] Robinson CD, Gonzalez-Feliciano A, Mucci LA, Markt SC (2018). Smoking cessation among men following cancer diagnosis: a matched cohort study. J Cancer Surviv.

[23] Hackett RA, Moore C, Steptoe A, Lassale C (2018). Health behaviour changes after type 2 diabetes diagnosis: findings from the English longitudinal study of ageing. Sci Rep.

[24] Schneider KL, Andrews C, Hovey KM, Seguin RA, Manini T, Lamonte MJ (2014). Change in physical activity after a diabetes diagnosis: opportunity for intervention. Med Sci Sports Exerc.

[25] Williams K, Steptoe A, Wardle J (2013). Is a cancer diagnosis a trigger for health behaviour change? Findings from a prospective, population-based study. Br J Cancer.

[26] Xiang X (2016). Chronic disease diagnosis as a teachable moment for health behavior changes among middle-aged and older adults. J Aging Health.

[27] Hernandez EM, Margolis R, Hummer RA (2018). Educational and gender differences in health behavior changes after a gateway diagnosis. J Aging Health.

[28] Neutel CI, Campbell N (2008). Changes in lifestyle after hypertension diagnosis in Canada. Can J Cardiol.

[29] Kang YW, Ko YS, Kim YJ, Sung KM, Kim HJ, Choi HY (2015). Korea community health survey data profiles. Osong Public Health Res Perspect.

[30] Joinpoint Trend Analysis Software [Internet]. National Cancer Institute, Division of Cancer Control & Population Sciences. Available from: https://surveillance.cancer.gov/joinpoint/ Accessed 1 Jan 2020.

[31] Preventing noncommunicable diseases. [Internet]. Geneva: World Health Organization. Available from: https://www.who.int/activities/preventing-noncommunicable-diseases Accessed 1 Jan 2020.

[32] Bauer UE, Briss PA, Goodman RA, Bowman BA (2014). Prevention of chronic disease in the 21st century: elimination of the leading preventable causes of premature death and disability in the USA. Lancet..

[33] Glantz S, Gonzalez M (2012). Effective tobacco control is key to rapid progress in reduction of non-communicable diseases. Lancet..

[34] Ives SP, Heuschmann PU, Wolfe CD, Redfern J (2008). Patterns of smoking cessation in the first 3 years after stroke: the South London Stroke Register. Eur J Cardiovasc Prev Rehabil.

[35] Lawrence D, Mitrou F, Zubrick SR (2009). Smoking and mental illness: results from population surveys in Australia and the United States. BMC Public Health.

[36] Smith PH, Mazure CM, McKee SA (2014). Smoking and mental illness in the U.S. population. Tob Control.

[37] Aubin HJ (2009). Management of emergent psychiatric symptoms during smoking cessation. Curr Med Res Opin.

[38] Prochaska JJ (2011). Smoking and mental illness--breaking the link. N Engl J Med.

[39] Rodriguez-Cano R, Lopez-Duran A, del Rio EF, Martinez-Vispo C, Martinez U, Becona E (2016). Smoking cessation and depressive symptoms at 1-, 3-, 6-, and 12-months follow-up. J Affect Disord.

[40] Stepankova L, Kralikova E, Zvolska K, Pankova A, Ovesna P, Blaha M (2017). Depression and smoking cessation: evidence from a smoking cessation clinic with 1-year follow-up. Ann Behav Med.

[41] Parry CD, Patra J, Rehm J (2011). Alcohol consumption and non-communicable diseases: epidemiology and policy implications. Addiction..

[42] Baliunas DO, Taylor BJ, Irving H, Roerecke M, Patra J, Mohapatra S (2009). Alcohol as a risk factor for type 2 diabetes: a systematic review and meta-analysis. Diabetes Care.

[43] Samokhvalov AV, Irving HM, Rehm J (2010). Alcohol consumption as a risk factor for atrial fibrillation: a systematic review and meta-analysis. Eur J Cardiovasc Prev Rehabil.

[44] Lindblad BE, Hakansson N, Philipson B, Wolk A (2007). Alcohol consumption and risk of cataract extraction: a prospective cohort study of women. Ophthalmology..

[45] Birch J, Petty R, Hooper L, Bauld L, Rosenberg G, Vohra J (2019). Clustering of behavioural risk factors for health in UK adults in 2016: a cross-sectional survey. J Public Health (Oxf).

[46] Physical activity for patients with hypertension [Internet]. Geneva: World Health Organization; 2017. Available from: https://iris.wpro.who.int/bitstream/handle/10665.1/13561/9789290618010-hyp-mod5-eng.pdf Accessed 1 Jan 2020.

[47] Physical activity for patients with diabetes [Internet]. Geneva: World Health Organization; 2017. Available from: https://iris.wpro.who.int/bitstream/handle/10665.1/13561/9789290618089-diab-mod5-eng.pdf Accessed 1 Jan 2020.

[48] Schuch FB, Vancampfort D, Richards J, Rosenbaum S, Ward PB, Stubbs B (2016). Exercise as a treatment for depression: a meta-analysis adjusting for publication bias. J Psychiatr Res.

[49] Piotrowicz R, Wolszakiewicz J (2008). Cardiac rehabilitation following myocardial infarction. Cardiol J.

[50] Jo MW, Seo W, Lim SY, Ock M (2019). The trends in health life expectancy in Korea according to age, gender, education level, and subregion: using quality-adjusted life expectancy method. J Korean Med Sci.

